# FT-IR Analysis of *P. aeruginosa* Bacteria Inactivation by Femtosecond IR Laser Radiation

**DOI:** 10.3390/ijms24065119

**Published:** 2023-03-07

**Authors:** Irina Saraeva, Eteri Tolordava, Svetlana Sheligyna, Alyona Nastulyavichus, Roman Khmelnitskii, Nikolay Pokryshkin, Dmitriy Khmelenin, Sergey Kudryashov, Andrey Ionin, Andrey Akhmatkhanov

**Affiliations:** 1 P. N. Lebedev Physics Institute of Russian Academy of Sciences, 119991 Moscow, Russia; 2 N. F. Gamaleya Federal Research Centre of Epidemiology and Microbiology, 123098 Moscow, Russia; 3 Faculty of Physics, M. V. Lomonosov Moscow State University, 119991 Moscow, Russia; 4 Institute of Crystallography, Branch of the Federal Scientific Research Centre “Crystallography and Photonics”, Russian Academy of Sciences, 119333 Moscow, Russia; 5 School of Natural Sciences and Mathematics, Ural Federal University, 620000 Ekaterinburg, Russia

**Keywords:** Fourier-transform IR spectroscopy, IR laser inactivation, bacteria

## Abstract

We report the successful inactivation of *P. aeruginosa* strain by femtosecond infrared (IR) laser radiation at the resonant wavelengths of 3.15 μm and 6.04 μm, chosen due to the presence of characteristic molecular vibrations in the main structural elements of the bacterial cells in these spectral ranges: vibrations of amide groups in proteins (1500–1700 cm^−1^), and C-H vibrations in membrane proteins and lipids (2800–3000 cm^−1^). The underlying bactericidal structural molecular changes were revealed by the stationary Fourier-transform IR spectroscopy, with the spectral peaks parameters being obtained by Lorentzian fitting with the hidden peaks revealed by the second derivative calculations, while no visible damage to the cell membranes was identified by scanning and transmission electron microscopy.

## 1. Introduction

Infrared radiation (IR) of the medium and far range (MIR and FIR) has been used for a long time in the food industry for a wide range of applications: drying, enzyme inactivation, and pasteurization of various products [1]. The principle of pathogenic bacteria IR inactivation in products is based on denaturation of functional proteins in bacterial cells, which are responsible for their proliferation due to the destruction of hydrogen bonds that form the secondary and tertiary structure of these proteins [2]. The inactivating properties of IR radiation were studied earlier in a number of works [3,4,5,6]. It was shown in [3] that IR heating causes more damage to the cell walls in the exponential growth phase; for the cells that were in stationary phase, IR heating caused more RNA damage. The cell wall injuries caused a more prominent effect in microorganism inactivation: 3.9 log10 CFU/mL reduction in the exponential phase cells was reported, comparing to 1.8 log10 CFU/mL reduction in the stationary phase cells after the treatment with IR radiation (3.2 kW/m^2^ for 5 min). A significant bactericidal effect of Er:YAG laser radiation with a wavelength of 2.94 µm in root canal bacteria was also shown previously [7,8]. Moreover, treatment of *S. aureus* cells in the phosphate buffer with IR radiation for 20 min led to their heating up to 700 °C and caused damage to the cell wall, shrinkage of the cytoplasmic membrane, leakage of the cell contents, and mesosome disintegration [4]. The efficiency of the bacterial spores inactivation by IR radiation was raised with an increase in the water content within [5]. In the earlier mentioned works, in the context of the inactivation, IR radiation sources in the broad spectral range were investigated. Sources of IR radiation with several selected wavelengths in the region of maximal absorption of proteins and nucleic acids (≈6 μm) were proven to be more effective for microorganism inactivation, as experimentally demonstrated by Oduola et al. [6].

Food preservation is one of the most important problems nowadays in the food industry. IR-spectral characterization shows that the main components of food products strongly absorb IR radiation in 3 and 6 μm ranges [9], making these two wavelengths suitable in the drying of food products with high moisture content [10].

In our research, we used IR laser with the selected wavelengths of 3.15 μm and 6.04 μm to expose Gram-negative *P. aeruginosa* bacteria. These laser wavelengths were chosen due to the presence of the characteristic vibrations in the main structural elements of the bacterial cells in these spectral ranges: 1500–1700 cm^−1^-vibrations of the amide groups of proteins, corresponding to the laser radiation with the wavelength of 6 μm, 2800–3000 cm^−1^-vibrations of the C-H bond, the most common in all biopolymers, corresponding to the laser radiation with the wavelength of 3 μm. Previously, we have shown the strong excitation of hydrogen bonds in cells of *S. aureus* and *P. aeruginosa* bacterial cultures during their irradiation by low-intensity (~0.1–10 GW/cm^2^) ultrashort pulses in the mid-IR range (5–6.6 μm) in the region of their characteristic absorption bands of the proteins and lipids [11], and the threshold character of *P. aeruginosa* bacteria inactivation rate upon its treatment with femtosecond laser pulses with the intensities of several TW/cm^2^ [12]. In this work, we studied the effect of inactivation of pathogenic bacteria by femtosecond laser pulses in the IR range with wavelengths of 3.15 μm and 6.04 μm at significantly lower intensities. The comparative analysis of the IR laser irradiation effect was implemented with FT-IR spectroscopy data, TEM and SEM characterization along with XRD analysis, and the subsequent microbiological tests, confirming the antibacterial effect of the IR laser radiation.

From a fundamental point of view, the processes occurring at the molecular level during bacterial death are of a great interest. One of the most accurate methods used for such studies is Fourier IR spectroscopy, which is able to provide the information on the molecular composition of bacteria before and after the bactericidal exposure. The total area of the peak corresponds to the concentration of the functional group, and its width reflects the vibrational freedom. The Lorentz approximation of the peaks provides the necessary data. Besides the band position and its intensity, the third spectral parameter, the bandwidth, is also useful for the interpretation. The flexible structures will, thus, give broader bands than the rigid structures and the bandwidth is a measure of the conformational freedom. For the molecules that bind to the proteins, the restriction of the conformational freedom is a natural consequence of binding. The binding of a molecule may also confer enhanced rigidity to more distant parts of the protein.

## 2. Results and Discussion

### 2.1. CFU Count

IR femtosecond laser inactivation results in the partial inactivation of *P. aeruginosa* population. The results of the bacterial viability analysis show an intensity-dependent decrease in the CFU/mL number in the *P. aeruginosa* culture samples exposed to the laser radiation at the wavelength of 3.15 μm or 6.04 μm (Table 1). The observed decrease in the CFU/mL value by approximately one order of magnitude is similar for all the exposure times and wavelengths. A similar order of inactivation for both the wavelengths at the higher absorption of 6.04 μm radiation by the bacterial layer is probably due to the higher laser intensity of the 3.15 μm radiation, by approximately two orders of magnitude higher than for the 6.04 μm radiation. The number of CFU is dependent on the exposure time after the irradiation with 3 μm, its value decreasing with the exposure time. The CFU/mL value is possibly non-monotonous at the longer time periods of exposure.

### 2.2. SEM and TEM Characterization

**Figure 1:**
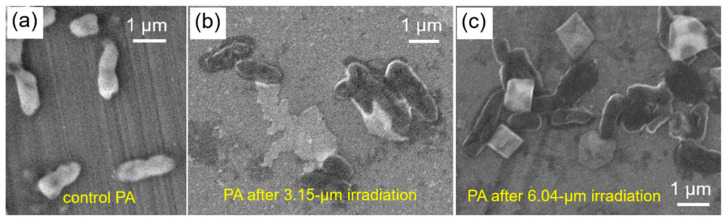
SEM images of *P. aeruginosa* dried on a *Si substrate*, before treatment (**a**), after 3.15 μm (**b**) and 6.04 μm (**c**) laser irradiations.

For SEM visualization, 6 µL drops of *P. aeruginosa* bacterial culture, prepared by the same method (see Section 3), were placed on the Si substrates and irradiated by the 3.15 μm or 6.04 μm laser radiation according to the procedure described there. The sample temperature was also monitored, not exceeding 25 °C. The resulting SEM images are shown in Figure 1.

**Figure 2:**
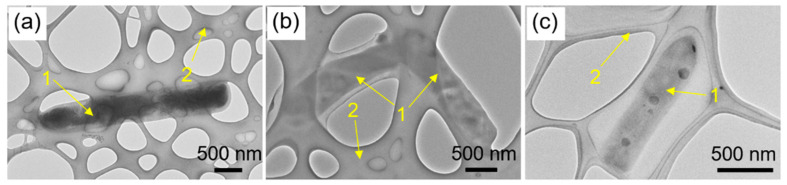
TEM images of *P. aeruginosa* cells, washed out from the CaF_2_ substrate, before treatment (**a**) and after the irradiation at wavelength of 3.15 μm (**b**) and 6.04 μm (**c**). 1—bacterial cells; 2—the carbon coating on the Au grid used in the TEM analysis.

For TEM analysis, the irradiated bacterial cells were washed out from the CaF_2_ substrates as follows. Each of the irradiated and control samples were put in the separate Eppendorf cuvettes, filled with 0.5 mL of distilled water, and shaken for 15 min. The washed down bacterial cells were put on carbon-coated Au grids and dried at room temperature (Figure 2).

The average size of *P. aeruginosa* cell equaled 1 µm, while its width was 0.7–1 µm (Figure 1). Treatment with IR radiation results in the formation of dark areas in the volume of the *P. aeruginosa* cells (Figure 2c) instead of the homogenously distributed cell contents in the untreated bacteria (Figure 2a). This may be attributed to the formation of protein coagulation due to the specific wavelength (6.14 µm) used for the amide region.

### 2.3. XRD Analysis

**Figure 3:**
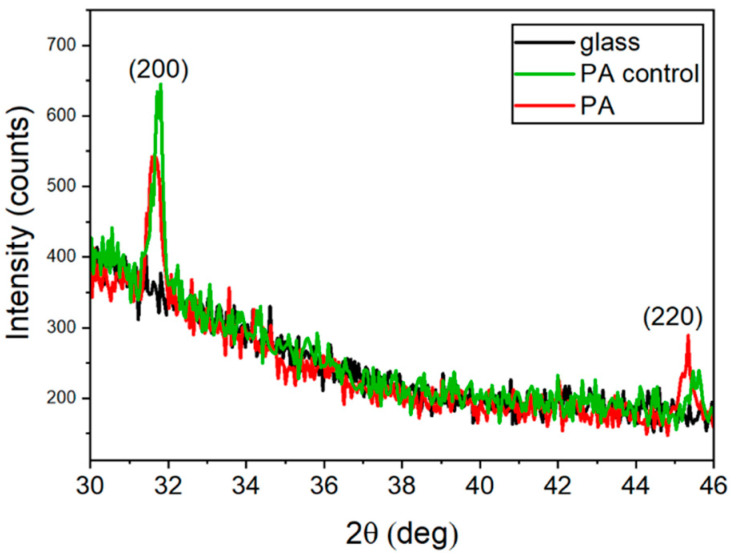
XRD spectra of *P. aeruginosa* (PA) on amorphous silica glass substrates: control (green lines) and after 6.04 μm irradiation (red line).

XRD analysis was implemented in order to monitor the possible crystallization in the bacterial cells. The XRD spectra show no evidence of crystallization, with the residual peaks corresponding to NaCl nanocrystals from the saline (Figure 3) [13]. Still, the intensity of the peak at 31.6 deg in the spectra of *P. aeruginosa* control samples decreases after the laser treatment, which indicates the possible degradation of the NaCl crystals.

### 2.4. FT-IR Spectral Analysis

**Figure 4:**
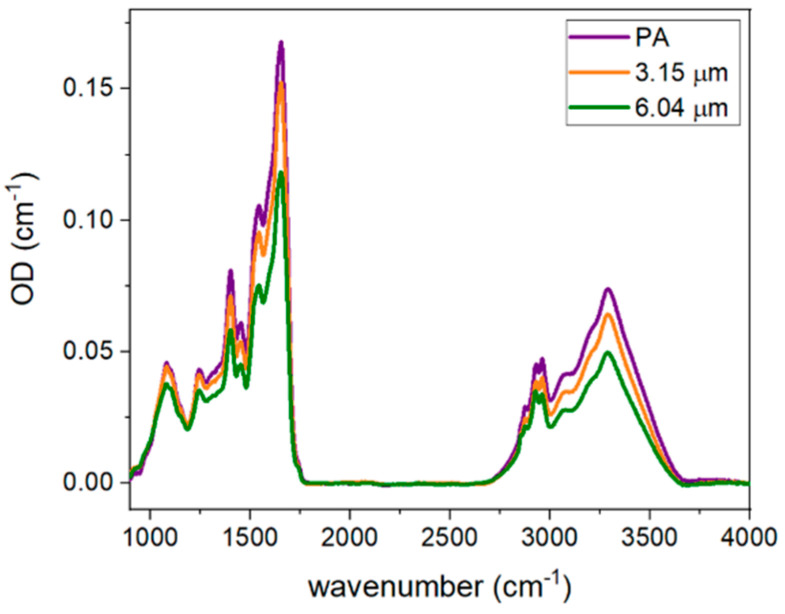
FT−IR spectra of *P. aeruginosa* (PA) on the CaF_2_ substrate before laser irradiation (control, violet lines), after 3.15 μm irradiation (orange lines) and 6.04 μm irradiation (green lines).

FT-IR optical density (OD) spectra after their baseline correction are shown in Figure 4. The overall intensity of the spectral peaks decreases after the IR laser irradiation at both of the used wavelengths.

**Figure 5:**
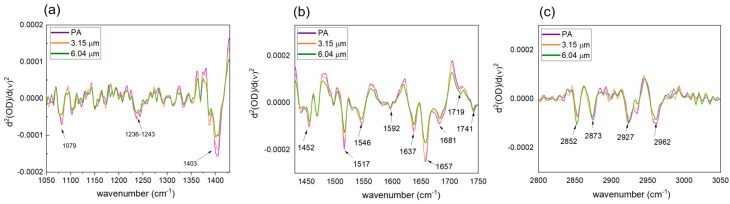
Selected regions of second-derivative FT−IR spectra of *P. aeruginosa* (**a**−**c**) on the CaF_2_ substrate before (purple line) and after laser irradiation at 3.15 μm (orange line) and 6.04 μm (green line) wavelengths. The main spectral peaks are marked by the arrows (see the description in the text).

Lorentzian fitting of the peak in the FT-IR spectra was implemented in Origin2019b 9.6.5.169 software with hidden peak detection by calculating second derivative (see Section 3 for details). Figure 5 shows the selected region of the second-derivative FT-IR spectra before and after the laser irradiation of *P. aeruginosa*.

The Lorentzian fitting results of the FT-IR spectra are shown in Table 2, with each cell containing three parameters: the first lines for each molecular vibrations being the vibrational frequency, the second, its bandwidths, and the third, its areas.

The residual water content is present in the bacterial cells, but the characterized peaks are attributed to the vibrations of amides and proteins, lipids, and fatty acids in the membrane, and the detected variations in these specific peaks were verified by the extensive literature data.

It is known that the outer membrane of the Gram-negative bacteria consists of lipopolysaccharides, containing phosphate and pyrophosphate groups, which render the cell surface negatively charged [14]. Therefore, the PO_2_ peaks at ~1080 cm^–1^ in *P. aeruginosa* spectra [15,16,17,18,19,20], which broaden after the 6.04 μm laser treatment (Table 2, Figure 5), may indicate the membrane damage. Fatty acids, also present in the membrane (peak at 2962 cm^−1^), exhibit strong narrowing and area decrease after the IR-laser treatment (Table 2, Figure 5), which indicates the decrease in relative motional freedom of molecules and the overall decrease in their concentration.

Asymmetric P=O stretching vibrations (1236–1240 cm^–1^) in phosphodiester, phospholipids, lipopolysaccharide, nucleic acids, ribose in bacterial membrane, nucleoid, and ribosomes exhibit the peak broadening after the IR-laser treatment (Table 2). The broadening of the peaks and the decrease in their area may indicate disorder and destruction of the corresponding molecular bonds.

COO¯-related peaks at 1403 cm^–1^ characterize peptidoglycans in the bacterial capsule [21]. Its shift to the lower wavenumbers and the decrease in the bandwidth, as well as in area values, after the irradiation of *P. aeruginosa* (Table 2) may indicate the roughening of the structure and the decrease in its content.

C-H (of CH_2_)-related peak at 1450–1452 cm^–1^ corresponds to molecular vibrations in lipids, which also form the cell membrane [21]. It exhibits the broadening with the unchanged area in *P. aeruginosa* (Table 2), which may indicate the disruption of the lipid structure.

The band at 1515–1517 cm^−1^ is associated with the ring vibration in the phenols of tyrosine side-chains [22]. In *P. aeruginosa,* tyrosine curates lipopolysaccharide synthesis, which is required for the drug-resistance development [23]. Therefore, its considerable broadening (almost two-fold) (Table 2) may indicate the hampering of the lipopolysaccharide synthesis, which is important from the point of view of drug-resistance study.

Amide II N–H, C–N vibrations illustrate the state of proteins in bacterial membranes, cytoplasm, flagella, pili, and ribosomes [21]. The peak broadening and its shift to lower wavenumbers after the 3.15 μm laser irradiation may indicate the destruction of the proteins (Table 2).

The amide I region represents the secondary structure of proteins in membranes, cytoplasm, flagella, pili, and ribosomes [21]. FT-IR spectral band analysis gives information about the structure and the environment of amino acid side-chains, bound ligands or cofactors, and the protein backbone can be deduced from the spectral band position, bandwidth, and area. Hydrogen bonds stabilize protein structure and are essential for catalysis, therefore, the disruption of these bonds leads to the protein destruction. The α-helical type of protein secondary structure is present in the spectra as a band at 1658 cm^–1^ [24], and in β-sheets as bands at 1637 and 1681 cm^–1^. The peak at 1658 cm^–1^ broadens after the IR-laser treatment at both the wavelengths, and its area decreases (more prominently at the 6.04 μm wavelength), which indicates the decrease in α-helices content after the IR irradiation (Table 2). The width of the amide I and II bands provides information on the vibrational freedom of proteins [18,25]. The proteins roughening may lead to the narrowing of the peak, which can be observed for peaks at 1637 cm^–1^ and 1681 cm^–1^ in *P. aeruginosa* (Table 2).

The peak corresponding to the CH_3_ symmetric vibrations of membrane proteins (2873–2875 cm^–1^) broadens after the IR-laser exposure, with the change being more pronounced after the 6.04 μm laser irradiation. The same dynamics are observed for CH_2_ asymmetric vibrations in lipids (Table 2).

CH_3_ asymmetric stretching in fatty acids is illustrated by the peak at 2962 cm^–1^, which narrows significantly after the IR exposure. Its area decrease is more pronounced after the 6.04 μm laser exposure for *P. aeruginosa* bacteria (Table 2).

## 3. Materials and Methods

*P. aeruginosa* bacterial strain was obtained from the collection of the N.F. Gamalei National Research Center for Epidemiology and Microbiology. The overnight culture (1 mL) was centrifuged, the supernatant was removed, then 1 mL of distilled water was added, and the resulting solution was shaken for 15 min. The resulting suspension was diluted by serial decimal dilutions to 10^5^ CFU/mL (colony-forming units per milliliter). The 6 µL drops of the resulting suspension were placed on the 2 mm thick CaF_2_ substrates (90% transmittance in the range of 0.15–9.0 μm).

**Figure 6:**
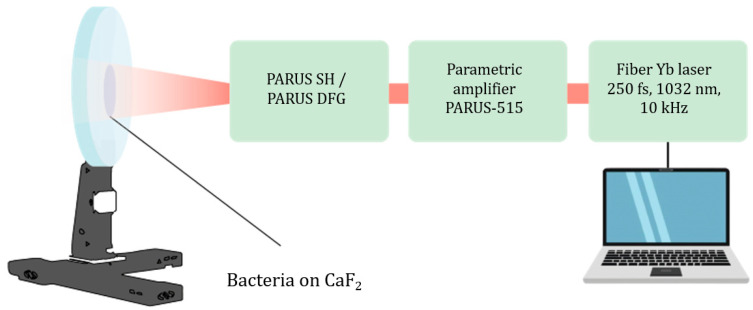
Experimental setup for the IR laser inactivation of the bacteria.

*P. aeruginosa* strain, placed on the CaF_2_ substrates and dried at room temperature, was exposed to the femtosecond (τ = 250 fs) laser pulses in the IR range with the central wavelengths of 3.15 µm and 6.04 µm for the time periods t = 3, 5, and 7 min. The output IR pulses were obtained by the subsequent parametric and difference frequency generation driven by the Yb^+^-doped fiber laser TETA (Avesta Project Ltd., Russia) at the wavelength of 1032 nm and repetition frequency f = 10 kHz (Figure 6). The laser pulse intensities equaled 1.2 × 10^8^ and 8 × 10^6^ W/cm^2^ at the selected 3.15 μm and 6.04 μm wavelengths, respectively (Figure 6). The bacterial irradiation was carried out by an unfocused laser beam, with the beam diameter on the sample surface equal to the 4 mm for the 3.15 μm radiation and to 5 mm for the 6.04 μm radiation. The temperature of the samples was monitored by means of the thermal imager UTi120S (Uni-T, Dongguan, China, temperature range: −20 °C ÷ 400 °C, display resolution: 320 × 240 pixels, thermal imaging pixels: 120 × 90, pixel size: 17 µm). The average temperature on the samples was around room temperature (25 °C), therefore, excluding the possible considerable global thermal effects in the bacterial inactivation.

After the laser exposure, all treated and control samples were placed in the separate sterile tubes with saline and vigorously shaken for 30 min. The resulting suspension was placed on a solid nutrient medium and kept in a thermostat for 24 h at 37 °C. After 24 h the bacterial colonies were counted to determine the CFU number, which was then recalculated to CFU/mL values. The obtained values were compared with the control samples that were not exposed to the laser radiation.

Surface topography of the bacteria was characterized by scanning electron microscope (SEM) TESCAN Vega (Tescan JSCo, Brno, Czech Republic) and by transmission electron microscope (TEM) Tecnai G12 (FEI company, Hillsboro, OR, USA) operating at 200 keV. For SEM imaging, a 5 µL drop of each sample was placed on a Si substrate and dried at room temperature. For TEM analysis, the aliquot of the liquid solution was placed on a carbon-coated golden grid.

XRD spectra acquired for the observation of the possible crystallization in bacteria, were obtained using a DR-02 RADIAN diffractometer (JSC Expertcentr, Moscow, Russia), with a Cu Kα X-ray source operating at the wavelength λ = 0.154 nm. Data were recorded for the 2θ range of 10–60° with a 0.05° step.

IR spectral measurements were performed in the range 800–3500 cm^−1^ with the use of a circular 2 mm diaphragm by means of a FT-IR spectrometer V-70 (Bruker, Billerica, MA, USA) in the vacuum camera. No correction for residual liquid or gas phase water absorption was made, which was found unnecessary due to identical conditions for the experiment and the samples’ post-characterization. The detected changes in the FT-IR spectra were not in any way caused by the residual water content and were, therefore, attributed to the changes in the amides and membrane lipids, proteins, and fatty acids. After measurement of IR transmittance, all spectra were converted to optical density (OD), normalized to the OD values of the substrate and corrected for the baseline. The spectra normalization was implemented by calculating the value of log(T_substrate_/T_sample_). The second derivatives were calculated in order to visualize the hidden peaks with its smoothing by Savitsky–Golay method (polynomial order 2, 10 points of window). The spectral peak analysis was implemented by Lorentzian fitting with the fixed values of their spectral positions.

## 4. Conclusions

In our work, we investigated the effect of resonant laser radiation at the wavelengths of 3.15 μm and 6.04 μm on *P. aeruginosa* bacterial strain. These wavelengths were chosen due to the coincidence with the characteristic vibrations of the main structural elements in the bacterial cells: vibrations of amide groups of proteins (1500–1700 cm^−1^) and vibrations of the membrane proteins and lipids (2800–3000 cm^−1^). SEM and TEM analysis illustrates the unchanged integrity of the cell membrane, therefore, suggesting the intermolecular change in bacterial cells. Analysis of the FT-IR spectra recorded for each sample was performed by Lorentzian fitting of the spectral peaks, with hidden peaks search implemented by second derivative calculation, and comparison of the peak parameters, such as peak spectral position (frequency), its bandwidth (full width at half-maximum), and area. The resulting data confirm the more pronounced effect of the 6.04 μm laser radiation on membrane proteins, lipids, and fatty acids in P. aeruginosa bacteria. The membrane lipopolysaccharides peak at ~1080 cm^–1^ in *P. aeruginosa* are also affected after the 6.04 μm laser treatment. The peak at 1236–1240 cm^–1^ in phosphodiester, phospholipids, lipopolysaccharide, nucleic acids, ribose in bacterial membrane, nucleoid, and ribosomes exhibit their broadening after the IR treatment at both these wavelengths. The peak at 1658 cm^–1^ broadens after the IR treatment, and its area decreases (more prominently at the 6.04 μm wavelength), which indicates the α-helices content decreasing after the IR irradiation.

## Figures and Tables

**Table 1:** CFU per ml values for *P. aeruginosa*: control (untreated bacteria), after irradiation at 3.15 μm and 6.04 μm.

	Exposure Time, min	*P. aeruginosa*, CFU/mL
Control	-	5·10^4^
After 3.15 μm	3 5 7	5·10^3^ 3·10^3^ 2·10^3^
After 6.04 μm	3 5 7	1·10^2^ 5·10^3^ 4·10^3^

**Table 2:** IR-peak parameters before and after 3.15 μm, 6.04 μm laser irradiation of *P. aeruginosa* bacteria. Each cell in the table contains three lines, corresponding to the peak frequency, bandwidth, and area. Errors within ±0.5 value.

Functional Groups	Frequency (cm^−1^) Bandwidth (a.u.) Area (a.u.)		
	Control	3.15 μm Exposure	6.04 μm Exposure
PO_2_ str (sym) of nucleic acids and phospholipids	1079.97 163.82 11.01	1079.97 123.37 6.76	1079.97 177.61 11.04
P=O str (asym) of phosphodiesters	1240.04 52.95 1.88	1240.04 53.28 1.67	1240.04 53.35 1.34
COO¯	1403.97 48.13 4.13	1402.04 45.25 3.29	1402.04 47.21 2.75
C-H def of CH_2_	1450.25 32.97 1.33	1450.25 40.19 1.61	1450.25 40.21 1.32
Tyrosine O-H, C-C, C-H	1517.75 38.78 2.13	1517.75 60.31 4.18	1517.75 64.62 3.28
Amide II N–H bend, C–N str of proteins	1544.75 48.19 3.95	1544.75 52.94 3.50	1542.82 58.79 3.06
Amide I β-pleated sheets	1637.32 51.26 7.21	1637.32 47.80 5.76	1637.32 48.61 4.59
Amide I α-helices	1658.53 34.06 4.43	1658.53 34.86 4.42	1658.53 34.45 3.33
Amide I β-pleated sheets	1681.67 29.08 2.84	1681.67 28.60 2.41	1681.67 27.87 1.82
C-H str (sym) of CH_2_ in fatty acids	2852.29 32.79 0.43	2852.29 22.25 0.18	2852.29 11.11 0.06
C-H str (sym) of CH_3_ CH_3_ str (sym) of mainly proteins	2873.51 27.65 0.51	2873.51 30.36 0.53	2875.43 69.43 1.27
C-H str (asym) in CH_2_ of mainly lipids	2927.50 34.59 1.19	2927.50 36.56 1.20	2925.58 42.66 1.43
C-H str (asym) in CH_3_ of fatty acids	2962.22 49.46 2.27	2962.22 46.98 1.85	2962.22 40.09 1.11

## Data Availability

The data presented in this study are available on request from the corresponding author.

## References

[1] Tyagi L., Sharma G.P., Verma R.C., Jain S.K., Murdia L.K., Mathur S.M. (2020). Infrared heating in food processing: An overview. IJCS.

[2] Elliott D.C., Elliott E.W. (2001). Biochemistry and Molecular Biology.

[3] Sawai J., Fujisawa M., Kokugan T., Shimizu M., Igarashi H., Hashimoto A., Kojima H. (1997). Pasteurization of bacterial spores in liquid medium by far-infrared irradiation. J. Chem. Eng. Jpn..

[4] Krishnamurthy K., Demirci A.L.I., Irudayaraj J. (2004). Inactivation of *Staphylococcus aureus* by pulsed UV-light sterilization. J. Food Prot..

[5] Hamanaka D., Uchino T., Furuse N., Han W., Tanaka S.I. (2006). Effect of the wavelength of infrared heaters on the inactivation of bacterial spores at various water activities. Int. J. Food Microbiol..

[6] Oduola A.A., Bowie R., Wilson S.A., Mohammadi Shad Z., Atungulu G.G. (2020). Impacts of broadband and selected infrared wavelength treatments on inactivation of microbes on rough rice. J. Food Saf..

[7] Schoop U., Moritz A., Kluger W., Patruta S., Goharkhay K., Sperr W., Georgopoulos A. (2002). The Er: YAG laser in endodontics: Results of an in vitro study. Lasers Surg. Med. Off. J. Am. Soc. Lasers Surg. Med..

[8] Folwaczny M., Mehl A., Aggstaller H., Hickel R. (2002). Antimicrobial effects of 2.94 μm Er: YAG laser radiation on root surfaces: An in vitro study. J. Clin. Periodontol..

[9] Hashimoto A., Yamazaki Y., Shimizu M., Oshita S.I. (1994). Drying characteristics of gelatinous materials irradiated by infrared radiation. Dry. Technol..

[10] Pawar S.B., Pratape V.M. (2017). Fundamentals of infrared heating and its application in drying of food materials: A review. J. Food Proc. Eng..

[11] Kompanets V.O., Kudryashov S.I., Totordava E.R., Shelygina S.N., Sokolova V.V., Saraeva I.N., Kovalev M.S., Ionin A.A., Chekalin S.V. (2021). Femtosecond infrared laser spectroscopy of characteristic molecular vibrations in bacteria in the 6-µm spectral range. JETP Lett..

[12] Kompanets V., Shelygina S., Tolordava E., Kudryashov S., Saraeva I., Rupasov A., Kovalev M. (2021). Spectrally-selective mid-IR laser-induced inactivation of pathogenic bacteria. Biomed. Opt. Express.

[13] Rabiei M., Palevicius A., Dashti A., Nasiri S., Monshi A., Vilkauskas A., Janusas G. (2020). Measurement Modulus of elasticity related to the atomic density of planes in unit cell of crystal lattices. Materials.

[14] Nikaido H., Nakae T. (1980). The outer membrane of Gram-negative bacteria. Adv. Microb. Physiol..

[15] Tang M., McEwen G.D., Wu Y., Miller C.D., Zhou A. (2013). Characterization and analysis of mycobacteria and Gram-negative bacteria and co-culture mixtures by Raman microspectroscopy, FTIR, and atomic force microscopy. Anal. Bioanal. Chem..

[16] Davis R., Mauer L.J. (2010). Fourier transform infrared FT-IR spectroscopy: A rapid tool for detection and analysis of foodborne pathogenic bacteria. Curr. Res. Technol. Educ. Top. Appl. Microbiol. Microb.Biotechnol..

[17] Melin A.M., Perromat A., Déléris G. (2000). Pharmacologic application of Fourier transform IR spectroscopy: In vivo toxicity of carbon tetrachloride on rat liver. Biopolym. Orig. Res. Biomol..

[18] Gorgulu S.T., Dogan M., Severcan F. (2007). The characterization and differentiation of higher plants by Fourier transform infrared spectroscopy. Appl. Spectrosc..

[19] Naumann D. (2001). FT-infrared and FT-Raman spectroscopy in biomedical research. Appl. Spec. Rev..

[20] Kamnev A.A., Tugarova A.V., Dyatlova Y.A., Tarantilis P.A., Grigoryeva O.P., Fainleib A.M., De Luca S. (2018). Methodological effects in Fourier transform infrared FTIR spectroscopy: Implications for structural analyses of biomacromolecular samples. Spec. Act. Part A Mol. Biomol. Spec..

[21] Quilès F., Humbert F., Delille A. (2010). Analysis of changes in attenuated total reflection FTIR fingerprints of *Pseudomonas fluorescens* from planktonic state to nascent biofilm state. Spec. Act. Part A Mol. Biomol. Spec..

[22] Zhu L., Qi H.Y., Kong Y., Yu Y.W., Xu X.Y. (2012). Component analysis of extracellular polymeric substances during aerobic sludge granulation using FTIR and 3D-EEM technologies. Bioresour. Technol..

[23] Zhao X., Lam J.S. (2002). WaaP of Pseudomonas aeruginosa is a novel eukaryotic type protein-tyrosine kinase as well as a sugar kinase essential for the biosynthesis of core lipopolysaccharide. J. Biol. Chem..

[24] Jackson M., Sowa M.G., Mantsch H.H. (1997). Infrared spectroscopy: A new frontier in medicine. Biophys. Chem..

[25] Garip S., Gozen A.C., Severcan F. (2009). Use of Fourier transform infrared spectroscopy for rapid comparative analysis of Bacillus and Micrococcus isolates. Food Chem..

